# Reliability and validity of the Adolescent Stress Questionnaire in a sample of European adolescents - the HELENA study

**DOI:** 10.1186/1471-2458-11-717

**Published:** 2011-09-23

**Authors:** Tineke De Vriendt, Els Clays, Luis A Moreno, Patrick Bergman, Germán Vicente-Rodriguez, Eniko Nagy, Sabine Dietrich, Yannis Manios, Stefaan De Henauw

**Affiliations:** 1 Department of Public Health, Faculty of Medicine and Health Sciences, Ghent University, De Pintelaan 185, 2 Blok A, B-9000 Ghent, Belgium; 2 Research Foundation - Flanders, Egmontstraat 5, B-1000 Brussels, Belgium; 3 Growth, Exercise, Nutrition and Development (GENUD) research group, Department of Physiotherapy and Nursing, University of Zaragoza, Domingo Miral s/n, 50009 Zaragoza, Spain; 4 Unit for Preventive Nutrition, Department of Biosciences and Nutrition, Karolinska Institutet, SE 141 57 Huddinge, Sweden; 5 Faculty of Health and Sports Sciences, Department of Physiotherapy and Nursing. University of Zaragoza, Ronda Misericordia 5, 22001-Huesca, Spain; 6 Department of Paediatrics, Medical Faculty, University of Pécs, Jzsef A 7, 7623 Pécs, Hungary; 7 Department of Pediatrics, Division of Clinical Nutrition, Medical University of Vienna, Währinger Gürtel 18-20, A-1090 Wien, Austria; 8 Department of Nutrition and Dietetics, Harokopio University, 70 E. Venizelou Ave., 17671 Kallithea, Athens, Greece; 9 Department of Health Sciences Vesalius, Hogeschool Gent, Keramiekstraat 80, B-9000 Ghent, Belgium

## Abstract

**Background:**

Since stress is hypothesized to play a role in the etiology of obesity during adolescence, research on associations between adolescent stress and obesity-related parameters and behaviours is essential. Due to lack of a well-established recent stress checklist for use in European adolescents, the study investigated the reliability and validity of the Adolescent Stress Questionnaire (ASQ) for assessing perceived stress in European adolescents.

**Methods:**

The ASQ was translated into the languages of the participating cities (Ghent, Stockholm, Vienna, Zaragoza, Pecs and Athens) and was implemented within the HELENA cross-sectional study. A total of 1140 European adolescents provided a valid ASQ, comprising 10 component scales, used for internal reliability (Cronbach α) and construct validity (confirmatory factor analysis or CFA). Contributions of socio-demographic (gender, age, pubertal stage, socio-economic status) characteristics to the ASQ score variances were investigated. Two-hundred adolescents also provided valid saliva samples for cortisol analysis to compare with the ASQ scores (criterion validity). Test-retest reliability was investigated using two ASQ assessments from 37 adolescents.

**Results:**

Cronbach α-values of the ASQ scales (0.57 to 0.88) demonstrated a moderate internal reliability of the ASQ, and intraclass correlation coefficients (0.45 to 0.84) established an insufficient test-retest reliability of the ASQ. The adolescents' gender (girls had higher stress scores than boys) and pubertal stage (those in a post-pubertal development had higher stress scores than others) significantly contributed to the variance in ASQ scores, while their age and socio-economic status did not. CFA results showed that the original scale construct fitted moderately with the data in our European adolescent population. Only in boys, four out of 10 ASQ scale scores were a significant positive predictor for baseline wake-up salivary cortisol, suggesting a rather poor criterion validity of the ASQ, especially in girls.

**Conclusions:**

In our European adolescent sample, the ASQ had an acceptable internal reliability and construct validity and the adolescents' gender and pubertal stage systematically contributed to the ASQ variance, but its test-retest reliability and criterion validity were rather poor. Overall, the utility of the ASQ for assessing perceived stress in adolescents across Europe is uncertain and some aspects require further examination.

## Background

Chronic stress is assumed to have role in the development of obesity by interacting with mechanisms underlying energy intake and expenditure, and stimulating visceral fat accumulation in favour of abdominal obesity [1]. Recently, further investigation of the facilitating effect of chronic stress on obesity was highlighted, particularly in adolescents [1]. Adolescence is characterized by remarkable plasticity, with fundamental physical, psychological and behavioural changes that require attention in terms of obesity research [2], and stress is considered to be inherent to this developmental stage [3]. As far as we are aware, there is no standardized methodology for the assessment of adolescent psychosocial stress, although stressor questionnaires (checklists or interview-based) are often used. Stressor checklists are self-reported questionnaires concerning the experience of stressful events by the respondent during a certain time period. They can be implemented on a large scale and are cost-effective [4]. Criteria for choosing a suitable checklist are a high validity, it must be relevant to its current time and relate to the scope of measurement (general or specifically focusing on certain stressors such as school stress), and be suitable for the target population. When using an existing checklist on a new population for which it was not originally developed, its validity should be considered.

Within the HELENA (Healthy Lifestyle in Europe by Nutrition in Adolescence) project [5], the association between stress and the onset of obesity in European adolescents was assessed. Owing to the lack of a recent stress checklist for use in European adolescents, the Adolescent Stress Questionnaire (ASQ), recently developed and validated by Byrne *et al*. for Australian adolescents, was utilized [6]. The ASQ was developed to address the requirement for systematic research examining adolescent stress in the early 21st century. Byrne and colleagues state that *'the ASQ is not a measure of symptomatic distress though it does assess subjective stressor load'*. The ASQ is a checklist containing 56 items covering a broad range of perceived adolescent stresses, with complete relevance to its current time. The items are statements concerning events or situations which adolescents could find stressful. These items require to be evaluated by the respondent in terms of the extent to which they were experienced as being stressful during the previous 12 months. The items were generated in adolescent focus groups, where adolescents brainstormed on the concerns and challenges associated with adolescence that had affected them or their peers. Using Principal Component Analysis, the items were attributed to 10 components or dimensions of adolescent stressor experiences (stress of home life, school performance, school attendance, romantic relationships, peer pressure, teacher interaction, future uncertainty, school/leisure conflict, financial pressure and emerging adult responsibility). These component scales were considered to be *'thematically meaningful within the existing body of theory and knowledge regarding the experience of adolescent stress' *[6] . The ASQ has a good construct validity (positive correlations with measures of anxiety and depression, and negative correlations with self-esteem), internal and test-retest reliability, and demonstrates consistent gender differences (girls report higher stress levels than boys), but poor correlations with age for certain stress dimensions [6]. The present study investigated the internal and test-retest reliability of the ASQ, its construct and criterion validity, and the independent contributions of socio-demographic characteristics (gender, age, pubertal stage and socio-economic status) to the variance in ASQ, when implemented in a multinational European epidemiological survey. If the ASQ was demonstrated to be a valid and reliable tool in a sample of European adolescents, it could be applied to identify important sources of adolescent stress in Europe and to investigate associations between adolescent stress and overall health, chronic morbidities (the development of obesity for example), mental well-being and other health-related outcomes.

## Methods

### Study design and population

The present study was implemented within the framework of the HELENA project [5]. The aim of the HELENA cross-sectional study (HELENA-CSS) was to obtain reliable and comparable data from a selected cohort of European adolescents concerning a broad variety of parameters related to nutrition, health, physical activity and fitness [5]. Adolescents were selected by random cluster sampling (all pupils from a selection of classes from all schools in 10 European cities), and stratified by geographical location, age and socio-economic status (SES). The sample size for the HELENA-CSS was calculated with a confidence level of 95% and ± 0.3 error, based on the variance in body mass index, and yielded a sample size of 300 in each city. A detailed description of the sample size estimation, the sampling and recruitment procedure of HELENA-CSS, is given by Moreno *et al*. [7]. In 10 European cities, 3865 adolescents (more than 300 per city) aged 12 to 17 years participated in the HELENA-CSS, and 3528 adolescents were eligible for inclusion (criteria included written informed consent, not participating in another survey, aged between 12.5 and 17.5 years, and data being available concerning an individual's gender, height and weight). Six of the 10 European cities included in the HELENA-CSS, participated in the stress module: Ghent, Stockholm, Vienna, Pécs, Zaragoza and Athens. Of the 2177 eligible adolescents from these six cities, a reduced sample size of 1240 adolescents participated in the stress sub-study. The sample was reduced because the stress module was optional and omitted when fieldworkers were constraint by time limits or logistically restricted.

The HELENA-CSS fieldwork was conducted between October 2006 and December 2007 throughout the whole school year except before, during or immediately after examinations, as this could have influenced the test results. The HELENA fieldwork consisted of a clinical and body composition examination, physical activity and fitness assessment, blood sampling and assessment of questionnaires [5]. The stress module encompassed the ASQ and measurement of salivary cortisol. These measurements were integrated in the fieldwork of the HELENA-CSS. Salivary cortisol was measured in a randomly selected sub sample of 50 pupils per city, but with the aim of having an equal distribution with regard to age and gender across the pupils who provided saliva samples. The present study was conducted according to the guidelines in the Declaration of Helsinki and the project protocol was approved by the local or national Ethics Committees of all the participating cities (the Ethics Committee of the Ghent University Hospital (Ghent, Belgium), the Regional Ethics Committee in Stockholm (Stockholm, Sweden), the Ethics Committee of the Medical University Vienna and the Vienna General Hospital (Vienna, Austria), the Regional Research Ethics Committee of the Medical Center in Pécs (Pécs, Hungary), the Research Ethics Committee of the Government of Aragon (CEICA, Zaragoza, Spain), and the Ethics Committee of Harokopio University (Athens)). All participants and their parents provided written informed consent for participation. Detailed information concerning the ethical/regulatory aspects and Good Clinical Practice within HELENA-CSS is described by Beghin *et al*. [8].

In addition to the HELENA-CSS study, a test-retest study of the ASQ was performed. A convenience sample of 55 voluntary adolescents (44% girls), with a mean age of 14.6 (± 1.1) years, was recruited in the region of Ghent. Criteria for participation included being aged between 13 and 17 years, having access to the Internet, non-participation in HELENA-CSS and a willingness to complete several paper-and-pencil questionnaires. Informed consent was obtained from the participants and their parents. Thirty-seven of the 55 adolescents completed the ASQ twice, with a test-retest interval of two weeks.

### Measurements

In the HELENA-CSS, a questionnaire was administered concerning socio-economic characteristics such as the number of cars and computers at home, internet at home and whether or not the individual had their own bedroom. Using these characteristics, the Family Affluence Scale was determined on the basis of a model developed by Currie *et al*. [9]. This model was adapted by replacing the item concerning 'holidays' (this was not assessed in HELENA) by 'internet at home'. The Family Affluence Scale indicated the socio-economic status (SES) on a scale of 0 (very low SES) to 8 (very high SES).

A clinical examination was performed during the fieldwork: birth date, gender and pubertal stage (according to the protocol developed by Styne [10]) were recorded and current medication use was reported.

Adolescent stress was assessed with the ASQ, developed and validated by Byrne *et al*. [6]. This ASQ assesses subjective stressor load, covering the broad domains of adolescent stressor exposure. The 56 items on this checklist were grouped into 10 stress component scales: stress of home life, school performance, school attendance, romantic relationships, peer pressure, teacher interaction, future uncertainty, school/leisure conflict, financial pressure and emerging adult responsibility. A complete list of the items and their allocation to the scales is presented in the 'Results'-section (Table 1). The items were ordered randomly (not by component scale) and preceded by a short introduction on how to fill in the ASQ. Respondents were asked to indicate on a 5-point Likert scale (1 = "not at all stressful (or is irrelevant to me)", 2 = "a little stressful", 3 = "moderately stressful", 4 = "quite stressful", 5 = "very stressful") how stressful these items had been to them during the past year [6].

**Table 1:** Item content and results of the 2nd-order confirmatory factor analysis of the Adolescent Stress Questionnaire (ASQ): standardized factor loadings of manifest variables and latent factors

	Standardized factor loadings	
	
ASQ scales with items	Manifest variables	Latent factors
Home life		.86
Disagreements between you and your father	.51	
Not being taken seriously by your parents	.51	
Little or no control over your life	.44	
Abiding by petty rules at home	.54	
Disagreements between your parents	.68	
Arguments at home	.69	
Disagreements between you and your mother	.66	
Lack of trusts from adults	.74	
Parents expecting too much from you	.67	
Parents hassling you about the way you look	.60	
Living at home	.51	
Lack of understanding by your parents	.77	
School performance		.81
Having to study things you do not understand	.58	
Teachers expecting too much from you	.55	
Keeping up with schoolwork	.56	
Difficulty with some subjects	.64	
Having to concentrate too long during school hours	.63	
Having to study things you are not interested in	.59	
Pressure of study	.70	
School attendance		.62
Getting up early in the morning to go to school	.36	
Compulsory school attendance	.78	
Going to school	.84	
Romantic relationships		.70
Being ignored or rejected by the person you want to go out with	.50	
Making the relationship with your boy/girl-friend work	.71	
Not having enough time for your boy/girl-friend	.74	
Getting along with your boy/girl-friend	.57	
Breaking up with your boy/girl-friend	.61	
Peer pressure		.85
Being hassled for not fitting in	.57	
Being judged by your friends	.67	
Changes in your physical appearance with growing up	.63	
Pressure to fit in with peers	.68	
Satisfaction with how you look	.56	
Peers hassling you about the way you look	.61	
Disagreements between you and your peers	.59	
Teacher interaction		.92
Disagreements between you and your teachers	.56	
Not getting enough timely feedback on schoolwork	.54	
Teachers hassling you about the way you look	.59	
Abiding by petty rules at school	.59	
Not being listened to by teachers	.68	
Lack of respect from teachers	.65	
Getting along with your teachers	.53	
Future uncertainty		.73
Concern about your future	.63	
Putting pressure on yourself to meet your future goals	.59	
Having to make decisions about future work or education	.71	
School/leisure conflict		.75
Not having enough time for fun	.74	
Not getting enough time for leisure	.78	
Having too much homework	.60	
Not enough time for activities outside of school hours	.71	
Lack of freedom	.62	
Financial pressure		.75
Pressure to make more money	.47	
Not enough money to buy the things you want	.77	
Having to take on new responsibilities with growing older	.60	
Not enough money to buy the things you need	.78	
Emerging adult responsibility		.85
Employers expecting too much from you	.50	
Having to take on new responsibilities with growing older	.57	
Work interfering with school and social activities	.60	

The original English version of the ASQ was translated twice into the local languages of the six participating cities (Dutch, Swedish, German, Hungarian, Greece and Spanish). These two independent translations were compared and a compilation of the best translations was carried out. Back translations were performed for quality control and local questionnaires were modified accordingly until agreement was obtained between the persons performing the translations. The ASQ was administered together with the other questionnaires of the HELENA study following a standardized procedure: questionnaires were completed in a classroom setting in total silence and fully supervised to avoid between-subject interaction. The answer categories 1 to 5 got the respective score (1-5). A score for each stress component scale was calculated by counting the scores of the items belonging to that scale. In addition, a stress summary score was obtained by adding the individual scores of all 56 items.

Baseline wake-up salivary free (BWSF) cortisol was measured in the adolescents as a biomarker for chronic stress to investigate associations with the ASQ. It is hypothesized that the ASQ scores of the component scales and the summary score are positively associated with BWSF cortisol. Cortisol is a main end product of the 'stress system'. Serum cortisol levels are the result of appraising all stress-inputs on the brain, coping and recovery from them, and are influenced by several neuro-endocrine and physiological pathways [1,11]. Unbound or free cortisol is also present in saliva [12], and is positively associated with acute [13] and chronic [12,14,15] stress. To reduce variance in cortisol levels due to diurnal variations in salivary cortisol [16], baseline (without stimulation) salivary cortisol was measured immediately after awakening. To account for intra-individual variability in salivary cortisol [17], awakening samples from seven consecutive days were collected. Saliva was sampled during the same week as the other HELENA measurements. Participants were subject to an oral introduction on how to take saliva samples (including time and conditions of sampling, demonstration of sampling procedure, points of attention to be addressed such as no eating, drinking or brushing teeth, and cooled storage of samples) and a detailed instruction sheet was provided.

A thorough protocol was composed in English explaining the procedure of saliva sampling to ensure standardization across the six cities. Saliva was collected with Salivettes^® ^(Sarstedt, Germany), providing stable samples at room temperature for a minimum of one week. The Salivettes^® ^were centrifuged at 2000 g for 10 min, and the filtrates were stored at -20°C. Before analysis, the samples were thawed and mixed. Salivary cortisol was measured using a modification of an unextracted radioimmunoassay method (Diasorin) for serum cortisol. Briefly, 200 μl saliva was pipetted into the coated tube and incubated with ^125^I cortisol for 45 minutes at 37°C. The modified cortisol assay had a measuring range from 0.5-30 μg/L and within- and between-run coefficients of variation of < 5% and < 10%, respectively. The cortisol concentration units from the analysis (μg/L) were converted into SI units (nmol/L) by multiplying the values with a conversion factor of 2.759 [18]. Only adolescents who had taken at least three saliva samples taken on awakening between 6 am and 8 am and with a cortisol concentration within the reference range of 3.0 to 54.9 nmol/L, as suggested by Groschl, Rauh & Dorr [19], were included in the validation analysis. For these adolescents, a mean value of their valid BWSF cortisol levels was calculated and used for further analyses.

### Data analysis

A total of 1240 eligible HELENA participants completed the ASQ, of whom 100 adolescents were excluded for the internal reliability and construct validity analysis of the ASQ, as they had more than four missing ASQ scales (n = 16) or 50% answers of 'do not apply' (n = 84), resulting in a study sample of 1140 adolescents. Comparison of the adolescents participating in the stress sub-study (N = 1240) with the total eligible HELENA sample (N = 2177) of the six cities revealed no significant differences in terms of gender distribution (*p *= 0.063) or age (*p *= 0.495).

Cronbach α-values of the stress component scales were calculated to ensure internal reliability. A test-retest reliability analysis was carried out by calculating the Intraclass Correlation Coefficient (ICC) between the scales of two assessments, two weeks apart, on a separate sample of 37 Belgian adolescents. A threshold of 0.8 for Cronbach α-values and ICCs was considered to indicate good internal reliability [20] and test-retest-reliability [21], respectively. Contributions of socio-demographic characteristics (gender, age, pubertal stage, SES) on the variability in ASQ scale scores and the summary score were investigated using Hierarchical Linear Models (HLM) with Restricted Maximum Likelihood Estimation [22], whereby 'city' was specified as subject grouping variable, and gender, age, pubertal stage and SES as fixed parameters.

A Confirmatory Factor Analysis (CFA) was performed to examine whether the original scale construct of the ASQ was confirmed by the data from the European adolescent population. The first-order model, with the individual items as manifest variables and the 10 component scales as first-order factors, demonstrated high mutual correlations between the factors (two thirds of the correlations were ≥ 0.6, data not shown). Therefore, a second-order CFA with Maximum Likelihood Estimation was executed with the summary score as the second-order factor. The error variances of the individual items were set as free parameters, while the variances of the latent factors were fixed at 1.0 in the model. The standardized factor loadings obtained represent the correlation between the observed variables and the extracted factors. Standardized loadings above 0.4 indicated an acceptable correlation and loadings above 0.5 indicated a good correlation; loadings below 0.4 were indicative of a poor correlation. Model evaluations were carried out using three 'goodness of fit' indices: χ^2 ^is reported as an absolute fit index, while Bentler's comparative fit index (CFI) and Root Mean Square Error of Approximation (RMSEA) are reported as comparative indices. For Bentler's CFI, a threshold of > 0.90 was considered to indicate a good fit [23] and for RMSEA, values < 0.05 or < 0.08 were representative of a good or acceptable fit [24], respectively.

For comparison of the ASQ with BWSF cortisol (criterion validity), 255 eligible participants provided saliva samples. Of these, 15 adolescents were excluded as they did not have a valid ASQ, 11 adolescents were excluded for taking oral contraceptives or steroids (these were the only reported medications documented to influence salivary cortisol levels [25]), and 29 adolescents were excluded for having no valid saliva samples (a minimum of three saliva samples taken on awakening between 6 am and 8 am and with a cortisol concentration within the reference range of 3.0 to 54.9 nmol/L were required). Data from 200 adolescents were used to assess the criterion validity of the ASQ. To investigate positive associations between the ASQ scores (scales and summary) and BWSF cortisol, linear regression analyses were performed with BWSF cortisol as dependent and with one of the ASQ scale scores or summary score as predictor (one model for each score), additionally controlling for pubertal stage, and this for boys and girls separately. Assumptions for performing linear regression analysis (independent observations, linearity, homoscedasticity, normality of residuals, non-collinearity between predictors) were verified and these were all met.

The CFA analysis was performed using SAS version 9.2; all other analyses were conducted using SPSS version 15.0.

## Results

Table 2 describes the study samples. Results of the internal and test-retest reliability of the ASQ are presented in Table 3. Only 50% of the stress component scales had Cronbach α-values of ≥ 0.8, indicating a moderate internal reliability of the ASQ scale construct. Test-retest reliability analysis of the ASQ resulted in ICCs lower than 0.8, with the exception of romantic relationships, demonstrating poor test-retest reliability between these measurements (Table 3).

**Table 2:** Participants of the validity study of the Adolescent Stress Questionnaire (ASQ)

	Athens	Ghent	Pecs	Stockholm	Vienna	Zaragoza	Total
Included participants with a valid ASQ							
Participants (n)	33	290	208	259	44	306	1140
Gender (% boys)	33.3	41.7	53.4	35.9	18.2	48.4	43.2
Age (mean ± SD))	13.2 ± 0.5	14.8 ± 1.2	14.4 ± 1.2	14.9 ± 1.2	15.9 ± 0.6	14.7 ± 1.1	14.7 ± 1.2
SES (mean ± SD)	2.9 ± 1.8	5.5 ± 1.5	3.9 ± 1.7	5.4 ± 1.4	5.3 ± 1.3	4.5 ± 1.5	4.8 ± 1.7
Pubertal status (%)							
Pre-pubertal (stage I)	6.1	0.3	0.0	0.4	0.0	0.0	0.4
Beginning pubertal (stage II)	27.3	3.8	1.4	8.9	0.0	1.6	4.5
Mid-pubertal (stage III)	57.6	12.1	19.2	28.2	0.0	5.9	16.2
Advanced pubertal (stage IV)	6.1	33.1	54.3	42.5	97.7	12.4	35.3
Post-pubertal (stage V)	3.0	47.6	24.0	12.4	0.0	75.8	39.7
Missing	0.0	3.1	1.0	7.7	2.3	4.2	3.9
Included participants with a valid ASQ and cortisol samples							
Participants (n)	27	39	30	34	20	50	200
Gender (% boys)	29.6	56.4	43.3	44.1	20.0	46.0	42.5
Age (mean ± SD))	13.2 ± 0.4	15.5 ± 1.0	13.5 ± 0.5	13.5 ± 0.4	16.1 ± 0.4	14.3 ± 0.6	14.3 ± 1.2
SES (mean ± SD)	2.6 ± 1.8	5.9 ± 1.3	3.3 ± 1.3	5.5 ± 1.3	5.5 ± 1.4	5.3 ± 1.4	4.8 ± 1.8
Pubertal status (%)							
Pre-pubertal (stage I)	3.7	0.0	0.0	0.0	0.0	0.0	0.5
Beginning pubertal (stage II)	29.6	0.0	3.3	23.5	0.0	0.0	8.5
Mid-pubertal (stage III)	59.3	7.7	23.3	55.9	0.0	8.0	24.5
Advanced pubertal (stage IV)	7.4	53.8	73.3	14.7	95.0	20.0	39.5
Post-pubertal (stage V)	0.0	35.9	0.0	2.9	0.0	72.0	25.5
Missing	0.0	2.6	0.0	2.9	5.0	0.0	1.5

SD: standard deviation; SES: socio-economic status

**Table 3:** Results from the internal and test-retest reliability analysis of the Adolescent Stress Questionnaire (ASQ) scores

ASQ scale	# items	Cronbach α	Test-retest ICC (95%CI)
Home life	12	0.88	0.69** (0.45-0.83)
School performance	7	0.81	0.60** (0.33-0.77)
School attendance	3	0.68	0.58** (0.32-0.76)
Romantic relationships	5	0.75	0.84** (0.71-0.91)
Peer pressure	7	0.82	0.69** (0.47-0.83)
Teacher interaction	7	0.80	0.68** (0.44-0.83)
Future uncertainty	3	0.68	0.64** (0.40-0.80)
School/leisure conflict	5	0.81	0.79** (0.63-0.89)
Financial pressure	4	0.73	0.69** (0.47-0.83)
Emerging adult responsibility	3	0.57	0.45* (0.15-0.68)
Summary score	56	0.95	0.89** (0.78-0.95)

ICC: intraclass correlation coefficient; 95%CI: 95% confidence interval

*p < .01; **p < .001

The independent contributions of socio-demographic variables to the variance in ASQ scale scores and the summary score were investigated using HLM (Table 4). Intercept-only models demonstrated that 'city' as grouping variable had a proportional explained variance of 6.7% for the summary score, while for the separate scales these proportional explained variances varied from 1.3% to 10.9%. Results of the HLM showed that mainly gender and pubertal stage systematically contributed to the variance of the majority of ASQ scales and the summary score: boys and those in a pre-, beginning-, mid-, or advanced pubertal stage (stage I to IV) reported lower stress scores compared to girls or those in a post-pubertal stage (stage V), respectively. Age and SES in general contributed to a lesser extent to the variance in ASQ scale scores and the summary score: age was only a significant negative predictor for stress from teacher interaction, peer pressure, and the summary score, while SES was a negative predictor for stress from teacher interaction and financial pressure.

**Table 4:** Contributions of socio-demographic characteristics to the variance in the ASQ scores in European adolescents

	HLM^a ^parameters									
	
	Intercept		**Sex**^**b**^		**Pubertal stage**^**c**^		Age		SES	
ASQ scale	Estimate	P	B	P	B	P	B	P	B	P
Home life	35.53	< 0.001	-3.46	< 0.001	-2.45	0.004	-0.23	0.442	-0.19	0.343
School performance	22.09	< 0.001	-1.95	< 0.001	-0.20	0.700	-0.06	0.763	-0.14	0.280
School attendance	8.25	< 0.001	-0.16	0.414	-0.21	0.401	-0.04	0.633	-0.10	0.115
Romantic relationships	12.00	< 0.001	-0.97	0.002	-0.89	0.029	0.00	0.997	0.03	0.786
Peer pressure	26.00	< 0.001	-2.34	< 0.001	-1.05	0.037	-0.56	0.002	-0.23	0.072
Teacher interaction	26.78	< 0.001	-0.17	0.657	-1.16	0.019	-0.64	< 0.001	-0.28	0.022
Future uncertainty	6.28	< 0.001	-0.98	< 0.001	-0.17	0.486	0.16	0.073	-0.06	0.366
School/leisure conflict	16.50	< 0.001	-1.17	< 0.001	0.16	0.708	-0.11	0.469	-0.06	0.569
Financial pressure	6.76	< 0.001	-0.35	0.149	-0.44	0.136	0.21	0.054	-0.25	0.001
Emerging adult responsibility	8.38	< 0.001	-0.06	0.744	-0.45	0.042	-0.13	0.097	-0.09	0.112
Summary score	182.63	< 0.001	-11.92	< 0.001	-8.30	0.021	-2.89	0.026	0.17	0.851

ASQ: Adolescent Stress Questionnaire; HLM: Hierarchical Linear Models; B: regression coefficient

^a ^Hierarchical Linear Models with 'city' as subject group variable

^b ^Reference category is girls

^c ^Reference category: pubertal stage V

The results of the second-order CFA are presented in Table 1. One item (item '*getting up early in the morning to go to school' *of school attendance) had a factor loading below 0.4, indicating a poor correlation with the respective factor (school attendance), while four items had acceptable factor loadings between 0.4 and 0.5. The majority of the items (51 out of 56) demonstrated high correlations with their component scale, with factor loadings > 0.5. The component scales correlated highly with the summary score, with factor loadings > 0.6. The models' absolute fit index χ^2 ^was 5112.11 (df = 1474, *p *< 0.001). The comparative model fit indices Bentler's CFI and RMSEA were 0.81 and 0.054, indicating a poor and acceptable fit, respectively, of the theoretical model of the ASQ in the data.

Results of the linear regression analysis for assessing the criterion validity of the ASQ are presented in Table 5. In boys, scale scores for school performance, peer pressure, future uncertainty and emerging adult responsibility were a significant positive predictor for BWSF cortisol. Results for the scale scores of home life and financial pressure and the ASQ summary score were borderline significant. In girls, none of the scale scores or the summary score came out as a significant positive predictor of the BWSF cortisol levels.

**Table 5:** Linear regression analysis for predicting the adolescent's BWSF cortisol with the ASQ scores and pubertal stage as predictors

	Model dependent: BWSF cortisol					
	
	Boys (n = 85)			Girls (n = 115)		
		
**Model predictor**^**1**^	β	t	p	β	t	p
Home life	0.203	1.896	0.062	- 0.083	- 0.890	0.375
School performance	0.246	2.293	0.024	- 0.025	- 0.269	0.788
School attendance	0.137	1.267	0.209	- 0.109	- 1.192	0.236
Romantic relationships	0.109	0.982	0.329	- 0.190	- 2.041	0.044
Peer pressure	0.248	2.343	0.022	- 0.057	- 0.614	0.541
Teacher interaction	0.108	0.988	0.326	- 0.006	- 0.065	0.948
Future uncertainty	0.223	2.078	0.041	- 0.056	- 0.610	0.543
School/leisure conflict	0.104	0.951	0.344	- 0.159	- 1.745	0.084
Financial pressure	0.198	1.840	0.070	- 0.122	- 1.289	0.200
Emerging adult responibility	0.262	2.440	0.017	0.076	0.795	0.429
Summary score	0.201	1.805	0.075	- 0.116	- 1.194	0.235

BWSF: Baseline Wake-up Salivary Free; ASQ: adolescent stress questionnaire; β: Standardized beta coefficients

^1 ^Besides the ASQ scale or summary score, the adolescents' pubertal stage was included as predictor in the regression models; only the regression coefficients and significance for the ASQ score as predictor are given.

## Discussion

The present study investigated the validity and reliability of the ASQ for assessing stress in European adolescents. The ASQ, originally developed for Australian adolescents, is documented to assess subjective stressor load and consists of 10 different stress dimensions covering a broad domain of adolescent stress experiences [6]. The reliability analysis in the present study indicated a moderate internal reliability of the ASQ scales, as five of 10 Cronbach α-values were > 0.8. The Cronbach α-values were lower than those in the validation study of the original ASQ carried out by Byrne *et al*. [6]. Test-retest ICCs of the ASQ scales in the present study were, with the exception of stress from romantic relationships, < 0.8, indicative of poor stability over time. They were lower than those reported by Byrne *et al*. [6], probably because of the greater within-measurement period (two weeks in the present study rather than one week in the study of Byrne *et al*. [6]) and the lack of power owing to a small sample size (37 in the present study compared with 105 in the study of Byrne *et al*. [6]).

The present study demonstrated that adolescent girls experienced higher stress levels than boys, confirming the gender differences in the ASQ as presented by Byrne *et al*. [6], and confirming previously reported gender differences in adults [26] and adolescents [27]. In addition, the present study demonstrated that pubertal stage was also a predictor for the ASQ, with the adolescents that are already in a post-pubertal stage of development having higher scores than those still in a pre-pubertal stage or in full pubertal development. This is in line with the hypothesis of Dahl and Gunnar that pubertal development is the driving force behind increasing stress sensitivity during adolescence [28] and with findings from Sumter *et al*. that the biological stress sensitivity increased with pubertal status [29]. The differential functioning of the ASQ according to the adolescents' gender and pubertal stage give strength to the validity of the ASQ. Age was no systematic predictor of the ASQ scores, suggesting that the ASQ functions similarly across the whole age range (12.5-17.5 years). This is not in agreement with observations of Byrne *et al*., where bivariate correlations with age were weak positive for five out of ten scales (significant correlations varied from 0.12 to 0.35) [6]. This might be indicative of differential scales functioning across cultures (see below). Surprisingly, no systematic association was evident between SES and the ASQ scores, conflicting with previous evidence that resource- and prestige-based indices of family SES were associated with adolescents' risk for uncontrollable and controllable negative life events, respectively [30]. These controversial results are probably because the ASQ assesses daily stressful situations or events, which have a lower impact on life and more frequent occurrence than the negative life events assessed in the study of Brady & Matthews [30].

The results of the second-order CFA suggest that the theoretical model of the ASQ fits moderately well with the observed data in the present population. The absolute goodness of fit index χ^2 ^was not indicative of a good fit. However, this fit index is widely recognized to be problematic as it is highly sensitive to sample size. The comparative fit indices, Bentler's CFI and RMSEA, were indicative of a marginally good or acceptable fit. The factor loadings of the majority of the observed items (51/56) were indicative of high correlations with the extracted factors. The factor loadings of the component scales on the summary score were higher (> 0.6), suggesting very good correlations between the scales and the summary score. The moderate construct validity could be explained by the fact that the nature of adolescent stress differs across culture boundaries. European adolescents may experience stress in a different way from Australian adolescents owing to differences in cultural background, education, living conditions, norms and standards. It could be hypothesized that the greater these differences, the lower the applicability of the original scale construct will be. This finding is in agreement with the concern outlined by Byrne *et al*. that *'the capacity of scales of adolescent stress to cross boundaries of culture is not yet well understood' *[6].

Linear regression analyses demonstrated that in boys, four out of 10 ASQ scale scores were found to be a significant positive predictor for their BWSF cortisol values, while in girls this positive association was not observed for any ASQ scale. These results indicate a rather poor criterion validity of the ASQ when compared with BWSF cortisol, especially in girls. A possible explanation for the weak or lack of distinct positive association between the ASQ and BWSF cortisol could be that the ASQ assesses only one aspect of the stress concept, namely the personal perception of stress from certain situations and events in adolescent life (so-called cognitive appraisal in the 'Stress and Coping Theory' developed by Lazarus and Folkman [31]), while the adolescents' coping mechanisms (defined as the second process in the 'Stress and Coping Theory' [31]) are not determined by the ASQ. Salivary cortisol is an indicator of Hypothalamic-Pituitary-Adrenal (HPA) axis activity and can be considered as a reflection of the adaptational outcome, which is the result of the appraisal of and coping with stress [13]. No controls for coping mechanisms could be used, and this could explain why increasing ASQ scores are not associated with increased salivary cortisol levels. This limitation of the ASQ restricts its utility to the assessment of cognitive appraisal of stress and not chronic stress, as a result of which its associations with stress-related health outcomes could be weakened or non-existent. Therefore, the information obtained with the ASQ should be complemented with information concerning previous lifetime stressful events (e.g. death of family members, sexual, psychological or physical abuse) as these are likely to influence the test results. Moreover, this additional information would complement the picture of the adolescents' chronic exposure to stress and provide more opportunities for investigating the potential role of chronic stress in the etiology of certain disorders. The lack of association between self-reported subjective measures of stress and cortisol measurements in biological samples has previously been denominated the 'lack of psychoendocrine covariance' [32]. Hellhammer *et al*. indicated that this phenomenon is not surprising given the complex interplay of neurobiological events that link perceived stress to HPA axis activity and the difficulties in assessing perceived stress [33].

In addition, in children with post-traumatic stress disorder (PTSD), long-term cortisol levels were no longer elevated as a reaction to trauma, but normalized [34]. The adolescents in the present study were not screened for previous development of PTSD or other severe traumas in their lifetime, and this could have reduced the expected positive association between perceived stress and cortisol levels. Therefore, future research is recommended to include all lifetime traumatic/stressful events in addition to focusing on a certain time period.

### Strengths and limitations

The multi-national character of HELENA-CSS and the stress sub-study, together with the strict standardization of the fieldwork across all European cities are great advantages of this project and make this project unique in its kind. The integration of the stress module in the HELENA-CSS had the advantage of investigating in-time associations between the experience of psychosocial stress and other health-related parameters (socio-demographic, clinical, food-related and physical activity parameters) conducted in HELENA. Unfortunately, the study also faces some limitations, which are specified below.

For feasibility reasons, the study population within the HELENA-CSS was determined on the basis of random cluster sampling. Inherent in cluster sampling, the standard errors for estimates are greater than for simple random samples, the so-called 'design effect' [35]. In view of these possible higher standard errors, it could be the case that some differences in ASQ scores between groups were not revealed.

Test-retest reliability analysis and criterion validity analysis were performed on a convenient sample of adolescents and are subject to limitations inherent to convenience sampling (e.g. non-generalization, and a potentially large and unmeasured bias). These data were collected for validation purposes only and the consequences of this convenience sampling are assumed to be minimal. In addition, the samples for these analyses were small and the lack of power could have attenuated the test results and contributed to the rather poor test-retest reliability and criterion validity.

Other limitations included the time limits for the fieldwork of HELENA (only a well-defined period could be spent in the schools) and the high load of the measurements. Owing to these logistic limitations, BWSF cortisol was measured as this could be carried out at home rather than during school hours. Alternative procedures and biomarkers, recently suggested to be promising biomarkers in the context of stress assessment, are the cortisol awakening response (defined by Clow *et al*. as *'the period of cortisol secretory activity in the first 45-60 min immediately post-awakening'*) [12,14,36], hair cortisol [37] and salivary α-amylase [38]. These alternative procedures and biomarkers for chronic stress may have led to better criterion validity. However, this large body of evidence was not available at the beginning of the present study-. In addition, several methodological challenges associated with the measurement of these alternative biomarkers have been documented [14,39], which would have been difficult to address in the school setting of the HELENA project. Previously, several methodological difficulties related to salivary cortisol assessment have been addressed in the literature, which are hard to control in epidemiological surveys [25,40]. In the present study, researchers experienced some difficulties in collecting and analysing salivary cortisol. For instance, it was impossible to standardize the time of arousal and therefore sampling time, a factor that is documented to influence cortisol values [41]. This was partly controlled by using samples taken on awakening between 6 am and 8 am. The adolescents' compliance was also an issue of concern, as it might have been weakened by the high burden of measurements in HELENA. Sometimes individuals forgot to take a sample and there was no guarantee of correct timing (immediate after awakening) when samples were provided. The issue of protocol compliance for saliva sampling has previously been outlined for children and adolescents [12] and in adults [40]. Samples were taken on different weekdays, another factor that influences cortisol values [41]. To what extent these methodological shortcomings have influenced cortisol values and further analyses in this study is difficult to evaluate. Therefore, it is recommended that salivary sampling protocols are strictly standardized in future research (a particular challenge in epidemiological surveys).

## Conclusions

The present study demonstrated an acceptable internal reliability and scale construct of the ASQ when it was implemented in a European adolescent sample. In addition, significant independent contributions of gender and pubertal stage to the variance in the ASQ were established. These strengths were counterbalanced by a poor test-retest reliability and criterion validity of the ASQ, probably because of methodological shortcomings, and failure to demonstrate effects of socio-economic status. Based on these shortcomings, the utility of the ASQ within an European adolescent sample is uncertain and further research concerning these aspects is required. In addition, supplementing the ASQ with questions concerning previous (severe) lifetime stressful events is recommended in order to have a more complete picture of adolescent chronic stress exposure.

## List of abbreviations

HELENA: Healthy Lifestyle in Europe by Nutrition in Adolescence; ASQ: Adolescent Stress Questionnaire; HELENA-CSS: Healthy Lifestyle in Europe by Nutrition in Adolescence Cross-Sectional Study; SES: Socio-Economic Status; BWSF: Baseline Wake-up Salivary Free; ICC: Intra-Class Correlation; HLM: Hierarchical Linear Models; CFA: Confirmatory Factor Analysis; CFI: Comparative Fit Index; RMSEA: Root Mean Square Error of approximation; HPA: Hypothalamic-Pituitary-Adrenal; PTSD: Post-Traumatic Stress Disorder

## Competing interests

The authors declare that they have no competing interests.

## Authors' contributions

TDV and SDH developed the design of the stress measurement. LM coordinated the total HELENA project on international level. LM, YM and SDH were involved in the design of the HELENA project and locally coordinated the HELENA project. TDV, PB, EN, SD, GVR organized the fieldwork and performed the data collection locally. EC was involved in manuscript drafting and coordinated the statistical analysis. All authors have reviewed the manuscript, gave input and made significant improvements.

## Pre-publication history

The pre-publication history for this paper can be accessed here:

http://www.biomedcentral.com/1471-2458/11/717/prepub
